# Neuroblastoma, a Paradigm for Big Data Science in Pediatric Oncology

**DOI:** 10.3390/ijms18010037

**Published:** 2016-12-27

**Authors:** Brittany M. Salazar, Emily A. Balczewski, Choong Yong Ung, Shizhen Zhu

**Affiliations:** 1 Department of Biochemistry and Molecular Biology, Mayo Clinic College of Medicine, Rochester, MN 55902, USA; Salazar.Brittany@mayo.edu; 2 Department of Molecular Pharmacology and Experimental Therapeutics, Mayo Clinic College of Medicine, Rochester, MN 55905, USA; Balczewski.Emily@mayo.edu (E.A.B.); Ung.ChoongYong@mayo.edu (C.Y.U.)

**Keywords:** neuroblastoma, big data, computational modeling, drug repositioning, networks, spontaneous regression, metastasis

## Abstract

Pediatric cancers rarely exhibit recurrent mutational events when compared to most adult cancers. This poses a challenge in understanding how cancers initiate, progress, and metastasize in early childhood. Also, due to limited detected driver mutations, it is difficult to benchmark key genes for drug development. In this review, we use neuroblastoma, a pediatric solid tumor of neural crest origin, as a paradigm for exploring “big data” applications in pediatric oncology. Computational strategies derived from big data science–network- and machine learning-based modeling and drug repositioning—hold the promise of shedding new light on the molecular mechanisms driving neuroblastoma pathogenesis and identifying potential therapeutics to combat this devastating disease. These strategies integrate robust data input, from genomic and transcriptomic studies, clinical data, and in vivo and in vitro experimental models specific to neuroblastoma and other types of cancers that closely mimic its biological characteristics. We discuss contexts in which “big data” and computational approaches, especially network-based modeling, may advance neuroblastoma research, describe currently available data and resources, and propose future models of strategic data collection and analyses for neuroblastoma and other related diseases.

## 1. Introduction

Many research approaches to pediatric cancers have been adapted from adult oncology research. However, due to manifold differences in cellular basis and molecular mechanisms between the adult and pediatric cancers, these approaches may need to be critically evaluated for and tailored to the pediatric oncology research context. A common model of oncology research involves identifying genetic alterations in a type or subtype of cancer, investigating their roles in tumorigenesis, progression, or drug resistance, and exploiting them or their pathways as therapeutic targets. However, many pediatric cancers have a striking paucity of recurrent mutations when compared to most adult cancers [1,2], and require new research perspectives to better understand their specific etiologies and vulnerabilities. Furthermore, just as pediatric cancers differ from adult cancers biologically, they also present unique obstacles to research. Unlike adult cancers, most pediatric cancers are exceedingly rare, and therefore subject to limited funding support and fewer potential subjects for clinical research [1]. As pediatric cancers can be more biologically and clinically heterogeneous than adult cancers, generating the study power necessary to understand the complexity of these pediatric diseases is a daunting task [1,3]. Hence, research methods involving large-scale “big data” approaches may hold the key to addressing these challenges.

New technologies, including next-generation sequencing, have recently enabled the rapid generation, analyses, and dissemination of large biological datasets. In oncology, specifically, high-throughput and large-scale “big data” methods have led to exciting and clinically relevant discoveries. For example, these technologies recently led to the identification of frequent mutations in anaplastic lymphoma kinase (*ALK*) gene in both familial and sporadic neuroblastoma cases; as a result, *ALK* inhibitors are currently in clinical trials with promising preliminary results [4,5,6]. Using neuroblastoma as a paradigm of pediatric cancer, the aim of this review is to broadly engage with a few important facets of “big data” in a pediatric research setting. It is the hope of the authors that the ideas proposed in this review can be a helpful starting point for neuroblastoma researchers or pediatric oncologists who are interested in augmenting their existing research with techniques derived from “big data” science.

## 2. Current State of Big Data Use in Neuroblastoma Research

Neuroblastoma is the most common extracranial solid tumor in children [7,8,9,10]. It accounts for 7% of malignancies diagnosed in children between the ages of 0 and 14 [9,11], and nearly 15% of pediatric cancer-related mortality [9]. Neuroblastoma arises from malignant neuroblasts descended from neural crest cells. During development, sympathoadrenal (SA) lineage of neural crest progenitor cells migrate to form the peripheral sympathetic nervous system (PSNS) in the body [12,13]. Transformation of undifferentiated SA progenitor cells results in tumor formation in the adrenal medulla in the abdomen and sympathetic ganglia along the sympathetic chains (Table 1). In the advanced stages, neuroblastoma cells metastasize widely to the bone marrow, bone, lymph nodes, liver, intracranial and orbital sites, lung, central nervous system, and skin (Table 1) [1,9] resulting in long-term survival rates less than 40%, even with intensive treatment [5]. Interestingly, a subset of neuroblastoma patients (stage 4S) presenting with widespread metastases usually spontaneously regress with minimal or no treatment (Table 1) [11,14,15,16]. Such diverse clinical phenotypes of neuroblastomas implicate its heterogeneous and complex biological bases and provide compelling rationale for further study.

### 2.1. Generation of Neuroblastoma Staging Systems and Their Importance in Guiding Big Data Generation and Downstream Analyses

The complexity of neuroblastoma development is staggering, thus necessitating new approaches not only to stratify patients, but also to guide big data generation and comparative analyses. Understanding disease etiology and progression requires collection of spatial data, as migration is implicated in disease onset and progression; temporal data, as development involves many time-stratified and -dependent phases; and other high-dimensional cell data, as the developing organism is a dynamic collection of simultaneously co-evolving cells and cell types. Research consortia devoted to staging the disease played an integral role in the field’s earliest attempts to collect such data and generate large-scale clinical neuroblastoma databases. The International Neuroblastoma Staging Series (INSS) (Table 1) stratifies neuroblastoma by risk level, tumor location and dissemination, and *MYCN* (V-Myc avian myelocytomatosis viral oncogene neuroblastoma derived homolog) amplification, using data from consortia in the United States, Europe, and Japan [15]. In part, INSS staging relied on surgical observations and extent of resection, and, therefore was subject to certain biases, however, it also recommends what tests to perform and which types of data to collect [18]. A more recent classification from the International Neuroblastoma Risk Group (INRG) Task Force involved collecting clinical data and biosamples from more than 8800 cases in North America, Australia, Europe, and Japan [16]. The INRG staging incorporates medical imaging data, adding significant data volume and variety, in staging and risk stratification to avoid the aforementioned surgical biases. Importantly, the INRG moves towards a more molecular and genetic classification of disease, incorporating such additional measurements as general tumor ploidy, chromosome 11q aberration, and classification of *MYCN* amplification as focal or diffuse in the tumor population [16]. The INRG Task Force exemplifies how careful curation of large datasets can revolutionize care [19], e.g., comparative analyses between the “4S” under INSS staging scheme with other neuroblastoma stages can advance our understanding how tumor regress.

### 2.2. Recent Discoveries Made Possible by Large-Scale Data Analyses

The evolution of neuroblastoma staging reflects the general trend of incorporating next-generation technologies into neuroblastoma research. Prior to high-throughput genomic sequencing, amplification of the oncogenic transcription factor *MYCN* was identified in 20% of neuroblastoma cases and associated significantly with high-risk disease and poor outcome [9,20,21,22,23,24,25]. With recent integrative genomic analyses, including whole-exome, genome, and transcriptome sequencing, genome-wide association studies (GWAS) and array comparative genomic hybridization (array-CGH) analyses [7,20,21,26,27,28,29,30,31,32], an increasing number of genetic and chromosomal alterations have been discovered. In our current review, we would like to briefly highlight key findings—in terms of genetic mutations and epigenetic alterations, chromosomal alterations, predisposition alleles, and aberrations in micro and long non-coding RNAs—generated from large-scale data analyses.

#### 2.2.1. Genetic Mutations and Epigenetic Alterations

Unlike adult cancers, which are characterized by many acquired somatic mutations, neuroblastoma exhibits surprisingly few recurrent mutations conserved across high-risk cases, as assessed in several large-scale sequencing studies [33]. Among those frequently mutated genes, activated mutations in the *ALK* kinase and loss-of-function mutations in the *PHOX2B* (paired-like homeobox 2b) transcription factor account for ~80% of hereditary neuroblastomas [9,28,33,34,35]. *ALK* acquired somatic mutations also have been reported in ~14% of all high-risk cases [33,35]. Recently, we and others showed the cooperative oncogenic effect of MYCN and activated ALK in prompting neuroblastomagenesis in both transgenic mouse and zebrafish models of neuroblastoma [36,37,38]; our results further confirm ALK is a key oncogenic driver, in collaboration with MYCN, in neuroblastoma pathogenesis. In addition, recurrent somatic mutations have also been found in the genes contributing to RAS-MAPK pathway activation, *PTPN11* (protein tyrosine phosphatase, non-receptor type 11), *NRAS* (neuroblastoma RAS viral oncogene homolog), *BRAF* (B-Raf proto-oncogene, serine/threonine kinase) and *NF1* (neurofibromin 1) [34,37]; chromatin remodeling, such as *ATRX* (α thalassemia/mental retardation syndrome X-Linked) and *ARID1A/B* (AT-Rich interaction domain 1A/B) [3,14,35,38]; Rac/Rho pathway regulation, such as *TIAM1* (T-cell lymphoma invasion and metastasis 1), *DLC1* (deleted in liver cancer 1), *ARHGAP10* (Rho GTPase activating protein 10), *ATRX*; and others, such as *MYCN*, *TP53* (tumor protein P53) [33,35] and so on.

#### 2.2.2. Chromosomal Alterations

Besides *MYCN* amplification (the hallmark of high-risk neuroblastoma), chromosomal alterations—specifically copy number variants at 1p, 11p, 2p, 3p, 4p, 6p, 6q, 1q, 11q and 17q—have been identified using array-CGH and single nucleotide polymorphism (SNP) analyses [3,5,36,37,39,40,41,42,43]. Loss of heterozygosity of chromosomes 1p and 11q are associated with increased disease severity, with loss of 11q notably being inversely associated with *MYCN* amplification [3,5]. Chromosome rearrangements resulting in an increased activation of *TERT* (telomerase reverse transcriptase) have recently been identified in ~23% of high-stage neuroblastoma with very poor prognosis but no *MYCN* amplification [44]. Structural defects caused by chromothripsis have been found to recurrently affect *ATRX*, *ODZ3* (odd oz/ten-M homolog 3 (*Drosophila*)), *PTPRD* (protein tyrosine phosphatase, receptor type D), and *CSMD1* (CUB and sushi multiple domains 1) in high-risk neuroblastoma [35].

#### 2.2.3. Predisposition Alleles

GWAS approach has been successfully applied to identify common polymorphic alleles that associate with a predisposition to neuroblastoma. The most common polymorphic alleles have been identified at the LIM-domain-only gene *LMO1* locus which is significantly associated with advanced stage neuroblastoma [39,45]. Very recently, we have found a polymorphism in the first intron of *LMO1* that can influence neuroblastoma susceptibility by affecting differential GATA transcription factor binding [46]. Other recently identified common SNPs which confer higher susceptibility to neuroblastoma include variants in *BARD1* (BRCA1-associated RING domain 1) [47], a non-protein coding *CASC15* (cancer susceptibility candidate 15) [48], *DUSP12* (dual specificity phosphatase 12) [41], *DDX4* (DEAD box polypeptide 4) [41], *IL31RA* (interleukin 31 receptor A) [41], *HSD17B12* (hydroxysteroid 17b dehydrogenase 12) [41], *LIN28B* (lin-28 homolog B) [36,49], *HACE1* (HECT domain and ankyrin repeat containing E3 ubiquitin protein ligase 1) [36], *FLJ44180* [46], *NEFL* (neurofilament, light polypeptide) [50], *NBPF23* (neuroblastoma breakpoint family, member 23) [37], and *TP53* [51].

#### 2.2.4. Implications of Micro and Long Non-Coding RNAs and Other Factors in Neuroblastoma

Functional genetic regulators of neuroblastoma development and progression also include micro RNAs (miRNAs) [52,53,54], long non-coding RNAs (lncRNAs) [25], alternative splicing programs [24], fragile sites [55] and genome-wide methylation [56,57,58,59,60,61,62,63,64,65]. miRNAs have been implicated in neuroblastoma metastasis [52], *MYCN* regulation [66,67], and cell differentiation [68]; thus they may serve as novel therapeutic targets, especially in high-risk, *MYCN*-amplified patients [53,69,70]. For example, Das et al., created a novel method of analyzing DNA methylation and miRNA expression, and were able to identify a panel of epigenetically regulated miRNAs that contribute to disease pathogenesis [54]. Zhang et al., found that *MYCN* directly regulates the expression of key splicing factors *PTBP1* (polypyrimidine tract binding protein 1) and *HNRNPA1* (heterogeneous nuclear ribonucleoprotein A1) in high-risk neuroblastoma, leading to increased cell proliferation and poor overall survival [24]. Long noncoding RNA—*NBAT-1* (Neuroblastoma Associated Transcript 1)—has been found to epigenetically down-regulate tumorigenic factors and promote differentiation of tumor cells [25]. Both miRNAs and lncRNAs have been implicated in cell differentiation and development [71,72,73], further emphasizing the importance of the developmental context of this disease. There are a number of databases where information on lncRNAs [74,75,76,77] and miRNAs [78,79] and disease associations can be found (see also Table 2).

### 2.3. Unique Features of Neuroblastoma Development, Metastasis, and Regression Warrant Further “Big Data” Analysis

#### 2.3.1. Embryonal Tumor Derived from Aberrant Neural Crest Development

While the roles of genetic components are important for understanding and treating cancers, they offer insight into just one of the potential mechanisms of disease pathogenesis. Since neuroblastoma occurs so early in life, and usually only early in life, the developmental context of disease development must be considered as well. A model of pediatric cancers described by Marshall et al., suggests that pediatric cancers can be linked back to some defect in signaling crucial to embryonic development [10]. Although cell death is a necessary part of embryonic development as many more cells are being produced than are actually needed for growth and organogenesis [10], neuroblastomas may derive from neuroblasts that persist despite apoptotic and cell cycle arrest signals, and subsequently adopt tumorigenic phenotypes [10].

Neural crest cells form between the surface ectoderm and developing neural tube during neurulation [13,90]. They then migrate and differentiate to form many diverse cell types and tissues, such as the neurons and glia of the enteric, sympathetic, and parasympathetic nervous systems, skeletal and connective tissues in the face, pigment cells, and the adrenal medulla [10,13]. Throughout this migration neural crest cells ubiquitously express *MYCN*, which induces them to proliferate, expand, and maintains stem cell characteristics [90,91,92,93]. Therefore, the normal function of *MYCN* is remarkably similar phenotypically to the abnormal function of tumors. Usually, expression of *MYCN* is stopped by signals that induce differentiation of neural crest cells into neuroblasts and other derivatives and is rarely expressed in fully differentiated cells [21,23,64,93,94,95,96,97]. If cells do not differentiate, because of excessive expression of *MYCN*, they generally undergo apoptosis [10]. Neuroblastoma may develop when neuroblasts evade this cell death signaling (e.g., through loss of *CASPASE-8* or activation of *ALK*) [90,98,99,100,101,102], and subsequently adopt tumorigenic phenotypes.

While *MYCN* amplification is the most common genetic event associated with high-risk neuroblastoma, over half of high-risk patients don’t harbor this alteration [9]. As such, alternative mechanisms underlying neuroblastoma development could involve pathways that are independent of *MYCN* signaling; this gap in knowledge may potentially be addressed through the use of computational strategies. In addition, signaling and interactions between neuroblasts and other cell types may be of additional interest, since paracrine interaction within the somites, neural tube, notochord, and dorsal aorta are vital for normal neural crest cell specification, migration and differentiation [13,90]. Alterations in the microenvironment, especially signaling from other types of cells surrounding neural crest could lead to its abnormal development, which might not be clearly identified by examining neuroblastoma samples alone. This potential etiology can be investigated through recently developed computational techniques that model paracrine signaling, elucidate contributions of the tumor microenvironment to disease, and propose treatments targeting the tumor-stroma interface [103,104].

#### 2.3.2. Wide-Spread Metastasis in High-Risk Neuroblastomas

Since 90% of cancer mortality results from metastatic lesions [105] and over 50% of neuroblastoma patients especially those are older than 1 year of age with *MYCN* amplification present wide-spread metastasis at diagnosis [106], it is important to understand the pathogenesis of neuroblastoma metastasis. Neuroblastoma metastasis occurs overwhelmingly in sites of normal neuroblast migration that leads to the development of multifocal hyperplasia [14]. Very few studies have compared single metastases to primary tumors [107], and none have looked at multiple metastases from single individuals. Despite the well-documented barriers to autopsy collection in pediatric patients [108,109], an important analysis would involve constructing phylogenies of multiple tumors such as genomic or transcriptomic data from single post-mortem individuals [110]. Additionally, in vivo animal models [111,112] with neuroblastoma cells labeled with fluorescent reporter genes can be useful to visually track the dissemination and metastases of tumor cell subpopulations to delineate the molecular mechanisms underlying neuroblastoma metastasis.

#### 2.3.3. Spontaneous Regression of Stage 4S Neuroblastomas

Spontaneous regression or remission has been reported in many pediatric and adult cancer types; one estimate has its pan-cancer incidence as 1/80,000 [113]. Conversely, neuroblastoma has a significantly higher rate: one study suggests 25% of neuroblastoma cases experience spontaneous remission or regression [114]. Of note, stage 4S cases, which comprise about 10% of neuroblastoma cases, generally result in complete remission with minimal or no intervention [9,14]. Therefore, the 4S subtype is a very valuable model in understanding—and potentially inducing—regression in other neuroblastoma classifications and other cancers. Brodeur et al., propose neurotrophin growth factor (NGF) signaling, immune intervention and epigenetic regulation as potential mechanisms for regression [14]. While differential gene expression has been detected between 4S and non-4S neuroblastoma subtypes, no significant different molecular signatures have been found between regressing and non-regressing individuals within the 4S subtype. Both of these interfaces warrant further study, possibly through experiments with in vivo animal models and large scale data. The development of novel computational tools to decipher context-specific molecular events that signify whether tumors remit because of certain factors, including tumor cell death or differentiation and intra- or extracellular signaling, can be a promising avenue for novel drug development [14].

## 3. The Promise of Available Big Data Resources in Neuroblastoma Research

The concept of “big data” is relatively new in many fields, including the biological sciences. Big data can be described as a catch-all term for situations in data science and analytics that have four Vs: volume, velocity, variety, and veracity [115]. In rare diseases like neuroblastoma, high data volume (petabyte scale) is not often seen, if ever, since there are few affected individuals from which to sample. There are, however, other aspects of the big data paradigm that are directly applicable. The use of animal models and cell lines that closely mimic the biology of human neuroblastoma reduces uncertainty and provides veracity. Data velocity—conventionally referring to streaming data requiring immediate analysis and decision-making—informs solutions to the technical challenges involved with high-throughput computing pipelines that rapidly integrate new information with shared data repositories and produce candidate hypotheses for immediate validation. Integrating disparate data types (e.g., genomic, imaging) from different models into a single analysis produce data variety that can be integral to unraveling the complexity of disease like neuroblastoma. Strategic evaluation of these characteristics will benefit any data-driven research setting.

**Figure 1:**
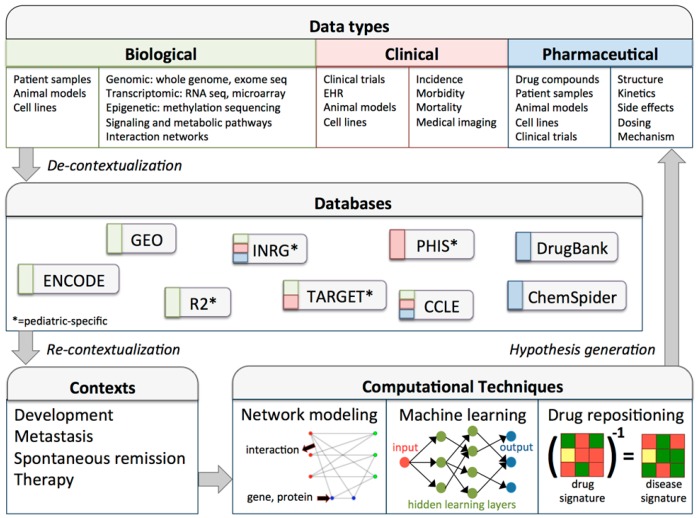
Data-driven research workflow for neuroblastoma or other pediatric cancers. The data types panel enumerates data sources (e.g., animal models) and types (e.g., RNA sequencing) for three categories of biological data. Following collection, data may be de-contextualized for inclusion in databases. De-contextualization involves properly formatting and annotating datasets, so that they are standardized, accessible, and useful for re-contextualization into new research contexts. Note that this workflow is iterative, and can therefore benefit from continued improvement of data infrastructure and data collections, development of more accurate and comprehensive data analysis tools, and advances in basic and translational research and therapeutic applications [35,118,119,120,121,122,123,124]. CCLE, cancer cell line encyclopedia; ENCODE, encyclopedia of DNA elements; GEO, gene expression omnibus; EHR: electronic health record; INRG, international neuroblastoma risk group; PHIS, pediatric health information system; R2, genomics analysis and visualization platform; TARGET, therapeutically applicable research to generate effective treatments.

Understanding the implementation—in addition to the qualities—of big data derived methods in biological research is critical. Figure 1 outlines a high-throughput workflow highlighting relevant data types, databases, and computational techniques that can be used by neuroblastoma researchers. Importantly, big data analytics is an iterative process, and interfaces between biological context and analytical technology must be continually considered. The processes of decontextualizing data from its native context for use in generalized databases, and subsequently re-contextualizing data for use in novel contexts are non-trivial. To execute these processes effectively, data must be strategically collected, contexts must be appropriate for data use, and computational techniques must be available to bridge the two.

### 3.1. Pitfalls in Clinical Data Collection

While optimal for research, high-throughput and high-fidelity pediatric cancer data can be difficult to collect. For one, pediatric cancers have lower incidence than adult cancers, and therefore fewer individuals to enroll in studies; in the US per 1 million individuals, neuroblastoma has an incidence of 21.1 and breast cancer, 123,700 [116]. While a large percentage of children with cancer are enrolled in clinical trials, certain pediatric groups—especially adolescents and low-income and non-white children—face barriers to participation [117]. Additionally, rates of autopsies performed on all patients in the US is low, occurring in about 5% of hospital deaths, but may be lower in pediatric patients due to the intrusive, insensitive perception of autopsies, unawareness of research studies, and the reluctance of physicians to ask bereaved family members [1,108]. Nevertheless, pathological samples gathered through autopsy can be important sources of high-quality data [109], and may even provide a small, positive experience for family members during their time of grief if the scientific value of the autopsy is emphasized [108]. We have assembled a table of databases where patient-derived data has been compiled (Table 2); though not comprehensive, it serves as a starting reference for researchers. The following sub-sections—animal models and cancers with related qualities to neuroblastoma—will describe alternate sources of potentially high-volume data that may serve to validate hypotheses generated by patient data, or explore the novel contexts supplied above.

### 3.2. Animal Models for Data Generation and Validation

Animal models provide several advantages to researchers; due to ease of use compared to human subjects and strong data-sharing communities, model organisms can be the basis for high volume and velocity data. For neuroblastoma, however, the distinctly important advantage of animal models is veracity in simulating the developmental context of neuroblastoma onset and progression. Unlike cell lines, which lack vascularity, immune involvement, paracrine signaling from other cell types, and other important factors contributing to tumor biology, animal models can better recapitulate human disease [112,125].

Neuroblastoma has several autochthonous or companion animal models, the most notable being *MYCN*- and *ALK*-expressing mice and zebrafish (Table 3) [98,102]. In the transgenic mouse or zebrafish model overexpressing *MYCN* in sympathoadrenal cells, animals developed tumors in the peripheral sympathetic nervous system, similar to neuroblastoma development in humans, and histological comparisons confirmed the similarity of these tumors to human tumors [102,126,127]. Combinations of gene alterations can also be investigated by crossing these models, such as between *MYCN* and *ALK*, *nf1* loss or *Caspase 8* loss [101,102,128]. Additionally, tumors from neuroblastoma patients can be xenografted into both zebrafish and mice, providing a way to capture the heterogeneity of human disease [94,129,130,131,132].

Zebrafish have an advantage over mouse models due to their small size, high fecundity, and ease of maintenance; additionally, their transparent embryos allow for easy observation of cancer progression and metastasis [127,133]. Conversely, mice more closely mimic human biology, and can be better analogs for investigating epigenetics [9], and drug therapies. Importantly, the Pediatric Preclinical Testing Program (PPTP) aims to expedite pediatric drug development by testing potential agents in mouse patient-derived xenografts (PDXs) [129]. While not close analogues of disease, *Drosophila melanogaster* models have been used to investigate the possible role of stemness in neuroblastoma [134,135]. Although in vitro models can be poorer proxies for disease than in vivo, cell lines generate high-quality and high-volume sequencing data and, in future, may better represent human disease through some developing technologies [136]. The Cellosaurus database [137] has a robust catalog of neuroblastoma-derived lines, as does Thiele’s 1998 review [138].

### 3.3. Similar Features between Neuroblastoma and Other Pediatric Solid Tumors

Grouping diverse cancers based on similar pathology, histology, or molecular biology can enrich oncological research efforts. A notable example involves the coordinated research efforts between ovarian and breast cancer researchers to identify and investigate the *BRCA1/2* (breast and ovarian cancer susceptibility protein 1/2) [139]. We believe similar approaches will be fruitful for neuroblastoma by increasing the volume of data available for analysis and provide insights for other pediatric cancers. Therefore, important axes for comparison between neuroblastoma and other types of pediatric solid tumors are summarized in Table 4.

## 4. Modeling Neuroblastoma-Derived Big Data

The rationale for modeling neuroblastoma with big data is to obtain new knowledge via hypothesis-free models without relying on prior biological knowledge. As neuroblastoma can be considered a complex trait (i.e., the disease results from interactions among multiple molecular factors), one of the key purposes of integrating multi-omics big data is to uncover genotype-phenotype interactions [155] that give rise to underlying neuroblastoma pathogenesis. The immense complexity and variability of big data is mirrored in the computational models used to extract meaning from it. Often the first line of analysis on biological big data involves statistical modeling and/or clustering to find significantly different or similar items. For genomic data, genome-wide association studies (GWAS) are an example of statistical interrogation of large datasets [156]. Clustering is more often used on RNA microarrays or sequencing to find groups of genes that co-express [157]. Finally, mathematical and rule-based modeling can simulate complex and unwieldy biological systems through a combination of experimental data and theoretical equations [158,159]. The remainder of the review will present strategies used to integrate big data and focus on three additional computational techniques—network modeling, machine learning, and drug repositioning—that are especially applicable to addressing the current research contexts in neuroblastoma described above.

### 4.1. Data Integration

Integrating different types or layers of big data offers many advantages and facilitates efforts to pinpoint genetic and molecular factors that play critical roles in regulating disease outcomes. Such integration compensates for missing or incomplete data coverage from technology that generates one single data type. In addition, when multiple sources of evidence from different data types point to a similar observation, confidence in findings is increased and false positives reduced. Furthermore, integrating different omics data allows for a more thorough and comprehensive understanding of how biological processes are regulated at different layers (i.e., from genetic mutations to epigenetic regulation, to transcription, to RNA processing, to protein synthesis, to protein modifications) and how malfunction at any of these layers causes neuroblastomas.

Although it is still a challenge to identify the best approach to integrate big data derived from different types and scales in a meaningful way, in general, there are two main strategies: meta-dimensional analysis and multi-stage analysis [160]. Meta-dimensional analysis integrates multiple datasets in a single study via three approaches: concatenating multiple data matrices from different omics data into one large input matrix before model construction (concatenation-based) [161]; transforming each data type into an intermediate form such as a graph matrix that represents a network before multiple data are combined (transformation-based) [162]; or generating different models by using different types of data as training sets before integrating into a final model, thus allowing independent analysis for each data type (model-based) [163]. Unlike meta-dimensional analysis, multi-stage analysis divides data analysis into multiple steps, capturing associations between enriched signals between different data types at each step. One example is genomic variation analysis where SNPs that associate with a phenotypic trait identified from GWAS are tested for their correlations with another omics datasets, such as transcriptomic data [163]. The associated SNPs are called expression quantitative trait loci (eQTLs) and genes associated with these eQTLs are in turn tested for correlations with the phenotype of interest.

### 4.2. Network-Based Modeling

Although network approaches have been applied to big data—most notably in social networks research [164]—their utility in biological research includes pathway analysis and integration of diverse data to model complex systems. We believe that network modeling algorithms—which have rarely been applied to neuroblastoma—are uniquely suited to provide insights into neuroblastoma development and treatment.

Network computation generally requires two types of data: (1) context- or phenotype-specific biological information (usually genomic, transcriptomic, metabolomic, or epigenomic); and (2) interaction information (usually curated protein-protein/gene-gene interactions, gene sets/signatures, or pathways) [165,166]. Recent expansion and more accurate annotation of protein-protein interaction (PPI) networks at both the genome and proteome scale [167] has facilitated discovery of novel functional crosstalk between genes. Thus far, PPI networks represent the most characterized biological network, although other type of networks, such as miRNA-gene networks [168], are being constructed. Computational modeling of cancer had been performed using PPI networks to decipher the effects of mutated genes on affected biological pathways [165,169]. Researchers can overlay biological data onto the network scaffold and investigate the structure or function of a given network (see Network panel of Figure 1 for an example of a gene interaction network). The databases shown in Figure 1 can be helpful sources for biological data to overlay onto curated networks; updated gene and protein interactions can be found in KEGG [170], BioGRID [171], and STRING [172], among others [173]. The commercial ingenuity pathway analysis and open-source network analysis tool (NeAT) online interfaces provide diverse network analyses in one integrated platform [174,175]. Additional platforms can analyze and visualize transcriptomic [176], metabolomic [177], or epigenetic data [178].

In addition to modeling approaches that use pre-constructed networks, other approaches use data to reverse-engineer networks. These approaches infer interactions between data through Pearson correlation coefficients, Bayes probabilistic models, and information-theoretic approaches such as mutual information [179]. The major advantages of reverse engineering methods are that no prior knowledge is required and novel cause-effect relationships can be inferred. Many of these methods have proven to possess the power to dissect gene regulatory modules in bacteria [180], yeasts [181], and human diseases [182], as well as specific biological process such as hematopoietic stem cell differentiation [183]. Reverse engineering approaches may be particularly suited to dissect novel key regulators driving neuroblastoma etiology and progression using large-scale multi-omics data. For example, we recently developed NetDecoder, a context-based network modeling tool that integrates both PPI network-based and reverse engineering-based approaches via a novel process-guided flow algorithm [176]. Our results showed that NetDecoder is capable of identifying genes that play critical roles under different disease contexts as evidenced by our breast cancer, dyslipidemia and Alzheimer’s disease case studies. For instance, we found the tumor suppressor gene *TP53* plays different regulatory roles in breast cancers versus dyslipidemia, indicating how the very same gene can cause different disease phenotypes under different pathological contexts. Context-dependent network modeling may be particularly important for understanding neuroblastoma etiology, given poor disease outcome may be shaped by *MYCN* amplification and *MYCN*-independent molecular mechanisms may drive higher risk among patients without *MYCN* amplification.

In addition, dynamical modeling using mathematical approaches, which facilitate modeling of cancer progression [184] and altered activities of signaling pathways [185], may prove useful in neuroblastoma modeling in the future. For example, dynamical modeling helped uncover the importance of phosphorylation events and effects due to gain or loss of protein-protein interactions in establishing novel pathway crosstalk. Using mass action law, our previous studies showed that dynamical modeling is a powerful approach to identify the “Achilles heel” of a cancer network, or genes sensitive to perturbations that may alter disease outcome [186]. Dynamical modeling may be applied to neuroblastomas, once key regulatory modules have been discerned via network-based modeling. For example, focusing on a particular pathway cascade such as how differential activities of Ras-MAPK cascade cause relapse [87] to decipher regulatory properties of neuroblastoma-specific molecular crosstalk may prove to be fruitful.

### 4.3. Machine Learning-Based Modeling

The term “machine learning” refers to a group of computational algorithms that can perform pattern recognition, classification, and prediction on novel data by “learning” from existing data (i.e., training set). In other words, machine-learning algorithms are mathematical mapping methods that aim to learn or uncover any underlying patterns (or features) embedded in the data. Machine learning has been applied in many areas of biological and medical sciences [187,188]. Currently, the most commonly used machine learning approaches in biology are support vector machines (SVMs) and artificial neural networks (ANNs). Recent advances in deep learning [189], specifically a more sophisticated ANN model with “deeper” neuron layers, spurred a number of applications in biology. For example, ANN can be used to predict sequence specificities of DNA- and RNA-binding proteins [190], effects of noncoding variants [191], alternative splicing [192], and quantitative structure-activity relationships of drugs [193] (Figure 1). These deep learning models have proven capable of outperforming other machine learning methods and identifying more complex features from data. Also, incorporation of deep reinforcement learning methods can facilitate improved performance in finding an optimal solution for a given task [194]. However, to achieve such levels of complexity, deep learning techniques generally require a higher volume of data and more computational time than other machine learning algorithms.

When using machine learning on biological big data, many different data types (genomic, transcriptomic, epigenomic, etc.) may be integrated into a single model. However, these data often have large dimension (e.g., whole exome sequencing has ~20,000 data points), and require many times more training data to build an appropriate model. Therefore, to make machine learning algorithms feasible for researchers, data collection can be minimized by reducing the dimension of input data. This dimensionality reduction can be done before or after data integration with Principle Component Analysis (PCA), or after data integration with feature selection algorithms [195]. Both of these strategies provide information about data sets by scoring variables on how much or little they contribute to a model.

Network modeling—specifically reverse-engineered networks—can be combined with machine learning. One method, Mode-of-action by network identification (MNI) reverse-engineers a network model of regulatory interactions using a training set of multidimensional biological data such as transcriptomics, proteomics, and metabolomics in order to identify genetic components and network modules that correspond to a cellular state. The method generates a directed graph relating the amounts of biomolecules to each other via a set of ordinary differential equations (ODEs). When transcriptomic data is used as training data, regulatory influences between genes can be inferred. MNI was recently applied to decipher causal genes responsible for driving tumor progression in ductal carcinoma in situ (DCIS), a non-invasive lesion of breast cancer. MNI identified *HoxA1* (homeobox A1) as the top candidate, and subsequent experimental work showed that in vitro silencing of *HoxA1* can revert the cancer phenotype [196].

CellNet is another network-based classification system with proven ability to classify cellular states based on their gene regulatory network (GRN) status [197,198]. CellNet was originally used to assess cellular states using their respective gene regulatory networks that act as major molecular determinants of cell type identity. GRNs were first reconstructed using a reverse engineering algorithm and then used as training data using Naïve Bayesian method. The study showed CellNet reverse engineered GRN-suggested neurons derived from directed differentiation of embryonic stem cells (ESCs) achieved a higher classification score than neurons derived from direct conversion from fibroblasts. CellNet also has the capability to infer causal genetic factors in driving cellular states such as *Pou2af1* (POU class 2 associating factor 1) and *Ebf1* (early B-cell factor 1) in B cells and *Foxa1* (forkhead box a1), *Hnf4α* (hepatocyte nuclear factor 4 alpha), *Cdx2* (caudal type homeobox 2) in hepatocytes. Thus, CellNet has the power to elucidate cell states via GRNs and identify potential regulators within GRNs that are responsible for cell state maintenance or transition. We anticipate that machine learning-integrated reverse engineering methods, such as MNI and CellNet, will hold the power to uncover causal genetic components that drive different stages of neuroblastoma.

### 4.4. Network-Guided Drug Discovery and Repositioning

Biological networks can also be efficient tools for identifying and evaluating drug targets. For example, Qin et al., used network flow algorithms to predict the sensitivity of cell lines to MAPK pathway inhibitors given as mono or combination therapy, based on copy number and mutational data for adult cancer cell lines in The Cancer Genome Atlas (TCGA) [199]. While the usual metric for selecting drug targets is gene and protein alteration [200], these network models can also identify genes and proteins that are necessary for disease progression or cell survival, but are not altered in any way. Given many of the altered genes in neuroblastoma, like *ALK*, have important roles in development of non-tumor cells [201], targeting other tumor-specific vulnerabilities could potentially reduce late effects from treatment.

The treatment of pediatric cancers is challenging due to the young age of the patients. The median age of children diagnosed with neuroblastoma is 17 months [9], therefore an ideal treatment would be non-invasive and minimize potential late effects. Treatment of neuroblastoma varies according to the stage of the disease (Table 1). However, children who receive treatment for aggressive disease can suffer from many treatment-related complications, such as hearing loss, cardiac toxicity, infertility, and increased risk of developing second cancers due to chemotherapy [11,202]. Because of these risks, more targeted and less harmful treatments are indicated. However, drug discovery in pediatric patients can involve many hurdles. For example, pediatric oncology patients are often excluded from clinical trials until drugs are shown to be effective at treating adult cancers [1]. This current method of drug discovery does not take into account the manifold differences between adult and pediatric cancers, such as the developmental context and low rate of recurrent mutations of pediatric cancers. Drug repositioning can leverage big biological, chemical, and pharmaceutical data to identify promising new neuroblastoma drug targets and combinations, and estimate their efficacy and safety in silico.

Prescribing drugs for treatment of other diseases beyond their previously approved uses is called drug repositioning or repurposing. This approach is especially useful in pediatric oncology research, since using previously pediatrically approved drugs in clinical trials can allow researchers to bypass Phase I safety trials. Several treatments for neuroblastoma have been repositioned, such as isotretinoin, originally an acne treatment, which is now a treatment for several other cancers, and an important component of multimodal therapy for advanced neuroblastoma patients [203]. Unlike many of the computational techniques above, the main object of performing drug repositioning is not to model or gain deep biological insights into neuroblastoma; rather, the approach seeks to rapidly bring treatments into clinic, by reducing the logistical burden of drug development and testing.

Drug repositioning algorithms follow a few broad computational paradigms: similarity measures, machine learning/mathematical modeling, and text mining. Similarity measures look for correlations between drug structure, functional category, mechanism of action, side effects, or transcriptomic or proteomic signatures. For example, if a drug shares a similar transcriptional profile with the inverse signature of aggressive neuroblastoma tumors (illustrated in the drug repositioning panel of Figure 1), it can be hypothesized that the drug could be effective. In addition, machine learning and mathematical models can pool many types of data, to find multi-dimensional similarity between drugs and diseases. One study examined the chemical structure, molecular activity, and side effects of drugs simultaneously to predict which drugs might behave similarly. Lastly, text mining methods look for associations between drugs and symptoms, genotypes, mechanism of action, or vice versa, within large publication databases to suggest novel drug-disease pairings [204].

A promising direction of drug repositioning is investigating the safety and efficacy of novel drug combinations, rather than single agents [205,206]. While additional safety trials are usually needed to test drugs in combination, combination therapies are, on the whole, more deleterious to cancer tissues than normal cells [207,208]. Of note, network-based drug repositioning algorithms have shown promise in repurposing drugs, both as monotherapies and in combination [209].

These drug repositioning strategies rely on robust clinical, genotypic, and drug structural data. A review by Ryall et al. [209], supplies an impressive list of such resources for drug repositioning, in addition to those listed in Figure 1. Nevertheless, many of these resources have scarce representation of pediatric drugs and diseases; for pediatric drug dosing, safety, and indications, researchers may access The Children’s Pharmacy Collective™, a novel online database, and the Harriet Lane Handbook, in print [210].

### 4.5. Future Modeling Challenges and Therapeutic Discovery

The ultimate goal of performing computational modeling on neuroblastoma using big data is to illuminate disease etiology and develop effective therapy. During the past few years, advances in network medicine [211] and systems pharmacology [212] have helped make strides toward this goal. However, as there are very rare recurrent somatic mutations detected in neuroblastoma, genetic approaches that aim to find druggable targets based on mutations alone are unlikely to yield fruitful drug discovery. For example, a mini-driver model of polygenic cancer etiology [213] proposes that many mutations found in cancers do not exhibit major driver effects but instead exert weak tumor-promoting effects. In addition, recent mathematical modeling work by Gatenby and colleagues suggests “never mutated” genes play a crucial role in cancer [200], indicating that most key genes may go undetected if only “mutation criterion” is applied to define cancer genes. Furthermore, epigenetic factors [214], RNA mis-splicing [215] or alternative splicing [216] and the presence of differentially expressed non-coding RNA species [217] have been reported to contribute to cancer etiology, and these processes are not necessarily affected by major mutations detected in most cancers. Such evidence challenges us to rethink how we could better model neuroblastomas at the network and systems level by integrating different types of big data. More sophisticated modeling approaches such as deep learning, which can integrate big data generated from patients and animal models, may be best suited to model the complexity of neuroblastoma etiology.

Another challenge that must be addressed: tumors frequently develop resistance to chemotherapeutics, rendering currently available drugs ineffective. Re-sensitizing tumors by modulating the network that regulates drug response may hold promise when tumors acquire resistance to existing drugs. Toward that end, our group recently developed Phenotyping Mapping (P-Map), a network-based tool that helps researchers to chart drug response networks [205]. Using anthracyclines and taxanes as case studies, we showed that it is possible to manipulate drug response phenotypes by perturbing key genes in a drug response network. Our study together with others (such as Chen et al. [218]) provide examples of efforts to rescue drug resistance via co-administration of compounds that modulate the activities of drug response networks.

## 5. Conclusions

Neuroblastoma is a common and complex pediatric cancer with extreme heterogeneity both at the clinical and molecular level [1]. Given many pediatric cancers share similar biology as well as research challenges, we seek to highlight the challenges and potential utility of using big data to further our understanding of neuroblastoma and related pediatric cancers and to develop effective drug treatments. The developmental context in which neuroblastoma arises appears to be particularly important, with many genes associated with aggressive disease (*MYCN*, *ALK*) driving cell proliferation and neuritogenesis [7,8,43,219]. Therefore, using animal models in which context is conserved is very important for generating more next-generation sequencing generated multi-omics data. Network models and drug repositioning can be leveraged to understand neuroblastoma and develop better treatment options.

The current review highlights the importance of collecting, annotating, and sharing data within the neuroblastoma research community, as well as the pediatric oncology communities. As new computational tools and perspectives arise, these data may be continually mined to generate new hypotheses and validate previously stated results. The biological complexity of disease must be matched by complexity of data and analysis in order to improve treatment at all stages of neuroblastoma.

## Figures and Tables

**Table 1:** Treatment and prognosis of neuroblastoma patients by risk group and staging. Prognosis is 5-year event-free survival [3,9,11,15,17]. *MYCN:* V-Myc avian myelocytomatosis viral oncogene neuroblastoma derived homolog.

Risk Group	International Neuroblastoma Staging System (INSS)	Tumor Localization	Characteristics	Treatment	Prognosis (5 Year Event Free Survival)
Low	1–2	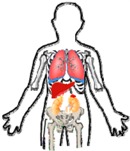	Stage 1—Small localized tumor, no *MYCN* amplification or chromothripsis.	Surgery, chemotherapy	>95%
			Stage 2—Localized tumor, some lymph node involvement, no *MYCN* amplification or chromothripsis.	Surgery, chemotherapy	
	4S	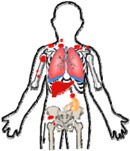	Stage 4s—Localized primary tumor, metastasis to liver, skin, bone marrow; diagnosed in infants <12 months of age. No *MYCN* amplification.	Observation	
Intermediate	3–4	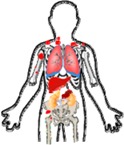	Stage 3—Tumor infiltrating across midline, regional or contralateral lymph node involvement, no *MYCN* amplification.	Surgery, chemotherapy	90%–95%
			Stage 4—Primary tumor, metastasis to lymph nodes, bone marrow, bone, skin, liver; diagnosed <12 months of age, no *MYCN* amplification.	Surgery, chemotherapy	
High	3–4	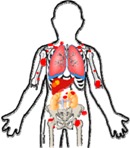	Stage 3—Tumor infiltrating across midline, regional or contralateral lymph node involvement, *MYCN* amplified.	Surgery, chemotherapy, radiotherapy, high-dose chemotherapy with autologous stem cell rescue, biologic and immunotherapeutic maintenance therapy, retinoids	40%–50%
			Stage 4—Primary tumor, metastasis to lymph nodes, bone marrow, bone, skin, liver; diagnosed >12 months of age, *MYCN* amplified.		

**Table 2:** A reference table of key resources for neuroblastoma big data.

Data Type	Database	URL	Reference
RNA expression	R2: Genomics Analysis and Visualization Platform	http://hgserver1.amc.nl/cgi-bin/r2/main.cgi	[80]
	Gene Expression Omnibus (GEO)	https://www.ncbi.nlm.nih.gov/geo/query/acc.cgi?acc=GSE49711, GSE16476, GSE65303, GSE49710, GSE22771, GSE16237, GSE3960, GSE45547, GSE53371	[43,81,82,83,84,85,86]
	ncRNA Expression Database (NRED)	http://nred.matticklab.com/cgi-bin/ncrnadb.pl	[75]
	Long Noncoding RNA Database v2.0 (lncRNAdb)	http://www.lncrnadb.org/	[76]
	LncRNADisease database (lncRNADisease)	http://210.73.221.6/lncrnadisease	[74]
	Human microRNA Disease Database (HMDD)	http://www.cuilab.cn/hmdd	[78,79]
	Therapeutically applicable research to generate effective treatments (TARGET): dbGaP	https://www.ncbi.nlm.nih.gov/projects/gap/cgi-bin/study.cgi?study_id=phs000467.v13.p6	[35,84,87]
DNA sequence	Neuroblastoma Cell Line Whole Exome Sequencing	https://www.ncbi.nlm.nih.gov/bioproject/PRJNA282395/	[87]
	GWAS Catalog	https://www.ebi.ac.uk/gwas/search?query=Neuroblastoma#study	[88]
	European Genome-Phenome Archive	www.ebi.ac.uk/ega/studies/EGAS00001000213	[35,89]

**Table 3:** Animal models of neuroblastoma. Table adapted from Zhu, 2016 [127], and expanded. ALK: anaplastic lymphoma kinase; CGH: comparative genomic hybridization.

Model	Type	Target	Tumor Location/Uses	Type of Data	Reference
Mouse	Transgenic line, *TH-MYCN*	Human *MYCN*	Thoracic, abdominal, metastasis to lung, liver, ovaries	CGH, histopathology	[126]
	Compound transgenic line, *TH-MYCN*, *TH-ALKF1174L*	Human *ALK F1174L* plus *MYCN*	Sympathetic ganglia or adrenals, locally invasive	Immunohistochemistry, transcriptomic	[98]
	Compound conditional transgenic line, *LSL-Lin28b*, *Dbh-iCre*	Mouse *Lin28b* plus *Cre*	Sympathetic ganglia or adrenals	Bioluminescence imaging, qRT-PCR, immunohistochemistry	[49]
	Xenograft in immune-deficient mice	Human *MYCN*-amplified primary tumor	Tumors engrafted into kidney capsule	Cell staining, qRT-PCR, testing NVP-BEZ235 treatment	[94]
	Compound conditional knockout line, *TH-MYCN*; *TH-Cre*, *Caspase 8*	Human *MYCN* plus loss of mouse *Caspase 8*	Sympathetic ganglia, metastasis to bone marrow	Immunohistochemistry, microarray, qRT-PCR	[101]
	Compound knock-in (KI), *TH-MYCN*; KI *Alk*	Human *MYCN* plus mouse *Alk*	Multifocal tumors, locally invasive	Immunohistochemistry, in vivo drug testing, transcriptomic	[99]
	Immune deficient, CB17SC-M *scid^−/−^*	Transplantation of human neuroblastoma cells	Subcutaneously injected tumor cells	In vivo preclinical drug testing	[129]
Zebrafish	Compound transgenic line, *DβH-MYCN*, *DβH-ALKF1174L*	Human *MYCN* plus human *ALKF1174L*	Adrenal, locally invasive	Immunohistochemistry, in situ hybridization, in vivo imaging	[102]
	Compound knockout line, *nf1a^−/−^*; *DβH-MYCN*	Human *MYCN* plus loss of zebrafish *nf1*	Adrenal, sympathoadrenal cells	In vivo imaging, immunohistochemistry, testing trametinib and isotretinoin	[128]
	Immunocompromised *rag2^E45^°^fs^*	Transplantation of zebrafish neuroblastoma cells overexpressing Human *MYCN* and *ALKF1174L*	Observation of tumor development and metastasis	In vivo imaging, flow cytometry, immunohistochemistry	[130,131]
	Transparent, *roy^−/−^*; *nacre^−/−^ “Casper”*	Transplantation of human tumor cells	Observation of tumor development and metastasis	In vivo imaging with resolution down to single cells	[131,132]
*Drosophila melanogaster*	Transgenic	Various targets involved in stem cell division	Understanding stem cell-like qualities of neuroblastoma tumors	Immunohistochemistry, tumor karyotyping, asymmetric cell division	[134,135]

**Table 4:** Similarities among neuroblastoma and other pediatric solid tumors. A cancer is marked pediatric if it has high incidence among all children with cancer, or has high pediatric incidence relative to adult. Small round blue cell tumors are cancers with a similar histologic appearance (highly nucleated, mesenchymal) that are often difficult to distinguish, especially in pediatric patients. If a subset of patients present with mutations or amplifications, a cancer is marked as being *MYCN* or *ALK* modified. While spontaneous regression is a rare event in all cancers, some experience slightly higher rates of treatment-independent full or partial regression of tumors and are included in the table as experiencing “spontaneous regression.” (*) Spontaneous regression cases of retinoblastomas may actually be benign retinomas, and therefore misclassified [96,97,140,141,142,143,144,145,146,147,148,149,150,151,152,153,154].

Cancer Type	Pediatric	Small Round Blue Cell Tumors	*MYCN*	*ALK*	Spontaneous Regression
Neuroblastoma	Yes	+	+	+	+
Retinoblastoma	Yes	+	+	+	
Medulloblastoma	Yes	+	+		
Glioblastoma	Yes		+	+	
Optic pathway glioma	Yes				+
Rhabdomyosarcoma	Yes	+	+	+	
Ewing sarcoma	Yes	+		+	
Melanoma	Rare			+	+
Small cell lung cancer	Rare	+	+		
Non-small cell lung cancer	Rare		+	+	
Wilms’ tumor	Yes	+	+		
Renal cell carcinoma	Yes			+	+

+ indicates that the corresponding cancer does exhibit the indicated trait.
